# Lightweight, Low-Cost Co_2_SiO_4_@diatomite Core-Shell Composite Material for High-Efficiency Microwave Absorption

**DOI:** 10.3390/molecules27031055

**Published:** 2022-02-05

**Authors:** Yifan Zhang, Rui Cai, Dashuang Wang, Kailin Li, Qing Sun, Yuntao Xiao, Hao Teng, Xiaohan Huang, Tao Sun, Zhaohui Liu, Kexin Yao, Yuxin Zhang, Pingan Yang

**Affiliations:** 1 College of Material Science and Engineering, Chongqing University, Chongqing 400044, China; zhangyifan@cqu.edu.cn (Y.Z.); Waloneds@sina.com (D.W.); likailin920809@163.com (K.L.); 20202732@cqu.edu.cn (Y.X.); 20192966@cqu.edu.cn (H.T.); 2 School of Automation, Chongqing University of Posts and Telecommunications, Chongqing 400065, China; crrrui@163.com; 3 College of Chemistry and Chemical Engineering, Chongqing University, Chongqing 400045, China; 20160902049@cqu.edu.cn; 4 Department of Military Facilities, Army Logistics University of PLA, Chongqing 401311, China; hxhshr@sina.com (X.H.); suntao_tju@126.com (T.S.); 5 Multi-scale Porous Materials Center, Institute of Advanced Interdisciplinary Studies, School of Chemistry and Chemical Engineering, Chongqing University, Chongqing 400045, China; zhaohui.liu@cqu.edu.cn (Z.L.); kexinyao@cqu.edu.cn (K.Y.)

**Keywords:** diatomite, Co_2_SiO_4_, electromagnetic wave absorption, nanomaterial

## Abstract

The porous and biomimetic cobalt silicate@diatomite (Co_2_SiO_4_@diatomite) was successfully synthesized by a two-step method, including the hydrothermal method and calcination to improve the electromagnetic wave absorption property. Different hydrothermal times were well-tuned for Co_2_SiO_4_@diatomite composites with different loadings of Co_2_SiO_4_. Interestingly, the Co_2_SiO_4_@diatomite composites (6 h, 25 wt%) had a smaller minimum reflection loss. Moreover, the minimum reflection loss (RL_min_) could reach −12.03 dB at 16.64 GHz and the matched absorber thickness was 10 mm, while the effective absorption bandwidth (EAB, RL ≤ −10 dB) could be 1.92 GHz. In principle, such findings indicate that Co_2_SiO_4_@diatomite nanocomposites could be a promising candidate for high-efficiency microwave absorption capability.

## 1. Introduction

In recent year, with the rapid development of electronic technology, electromagnetic (EM) pollution has become a non-negligible problem. EM pollution will interfere with the normal operation of electronic equipment [1] (such as the electronic equipment of hospitals and airports), which greatly impacts human production and life. Meanwhile, the damage of EM pollution to the human body must be, likewise, taken seriously [2]. Similarly, in the military field, the influence of electromagnetic waves (EMW) on the survival and attack of weapons also needs attention [3]. To solve these problems, EMW-absorbing materials (EMWAMs) are of great concern [3,4,5,6,7,8,9,10,11,12,13].

Carbon materials, such as carbon nanotubes, carbon fibers, porous carbon, and graphene [5,14,15,16], are one type of EMWAMs studied relatively early. Carbon materials are characterized by a low density, light weight, and good electroconductibility. Conductive polymers, such as polypyrrole [17] and polypyrrole@PANI [18], are of good conductivity, which induces the strong permittivity response by dielectric interaction to absorb EMW [19]. Metallic oxides, such as NiCo_2_O_4_ [20], Mn_3_O_4_ [21], CuCo_2_O_4_-CuO composites [22], and Co_1.29_Ni_1.71_O_4_ [8], have good magnetic loss characteristics, thus considered as excellent candidates for EMWAMs. However, carbon materials and conductive polymers have the disadvantages of impedance mismatching and narrow effective absorption bandwidth, and metal oxides suffer from gravimetric density and poor corrosion resistance [23]. To overcome these problems and broaden the application range of EMWAMs, diatomite, a biomimetic material with a unique 3D structure, has been noticed.

Diatoms are of unique siliceous cell walls that are micro-porous to nano-porous [24]. Diatomite is a bio-silica mineral obtained by geological deposition of the remains of single-celled diatoms [25]. Diatomite inherits this good characteristic of diatom and obtains extremely complex pore patterns. Therefore, diatomite with the intricacy and ordered pore pattern, the high surface area, and hollow and double-shell structure is considered to have good application potential in the field of EMWAMs.

Silicate nanomaterials are expected to be developed as absorbing materials due to their advantages of low cost, simple preparation, high thermal stability, and non-toxicity. Some transition metal silicates have both porous and layered structures, which makes them excellent candidates for EMWAMs. It is worth noting that in many transition metal silicates, cobalt silicate (Co_2_SiO_4_), as a magnetic material and in its theoretical capacitance, is of consideration [26,27,28]. Therefore, it has great application potential in EMWAM.

In this work, the diatomite-coated Co_2_SiO_4_ was synthesized successfully for the first time. Co-precursor@diatomite materials were obtained by the one-step hydrothermal method. Then, under high-temperature calcination at 900 °C, the Co-precursor@diatomite materials were gradually decomposed and reacted with diatomite to obtain Co_2_SiO_4_@diatomite materials. When the hydrothermal time was 6 h, the sample obtained by calcination had better EMW absorption performance than the other two samples. At 16.64 GHz, the sample of the reflection loss can reach −12.03 dB. The frequency falls within the Ku band used for satellite communications [29]. In a word, Co_2_SiO_4_@diatomite is a promising EMWAM.

## 2. Materials and Methods

### 2.1. Materials

Cobalt nitrate hexahydrate (Co(NO_3_)_2_·6H_2_O) and diatomite were purchased from Shanghai Aladdin Biochemical Technology Co., Ltd. (Shanghai, China) Ammonium fluoride (NH_4_F) and urea were purchased from ChengDu Chron Chemicals Co., Ltd. (Chengdu, China). All reagents were of analytical grade and used without any further purification.

### 2.2. Synthesis

**Figure 1:**
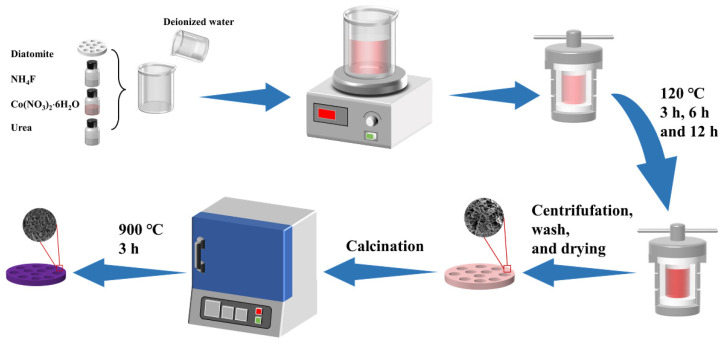
Preparation flow of Co_2_SiO_4_@DE.2.3. Characterization.

The synthetic process is shown in Figure 1. Co-precursor@diatomite was prepared via a hydrothermal method. Initially, 0.2325 g Co(NO_3_)_2_·6H_2_O, 0.08 g NH_4_F, and 0.275 g urea were dissolved into 50 mL of deionized water. The mixed solution was stirred magnetically for 30 min. Then, the solution and 0.1 g diatomite (DE) were transferred into a 100 mL Teflon-lined autoclave and heat-treated at 120 °C for 3 h, 6 h, and 12 h in a rotating oven. After the autoclave was cooled to room temperature, the solid product was centrifuged and washed by deionized water and ethyl alcohol. After drying at 60 °C, the Co-precursor@DE (−3, −6, −12) was obtained. Finally, Co-precursor@DE (−3, −6, −12) was calcined in a muffle furnace at 900 °C for 3 h to obtain Co_2_SiO_4_@DE (−3, −6, −12). 

The crystal structure and phase composition were characterized by an X-ray diffractometer (XRD, PANalytical X’ Pert Powder, Spectris Pte. Ltd., Shanghai, China). The composition and valence states of elements were characterized by an X-ray photoelectron spectrometer (XPS, ESCALAB250Xi, Thermo Scientific, Waltham, MA, USA). The microstructure, distribution, and composition of elements were characterized by field emission scanning electron microscopy (FESEM, Helios5-FIB, Thermo Scientific, Waltham, MA, USA), field emission transmission electron microscopy (FETEM, Talos F200S, Thermo Scientific, Waltham, MA, USA), and energy disperse spectroscopy (EDS). The changes in the sample quality during calcination were characterized by a thermal gravimetric analyzer (TGA, TGA2, METTLER TOLEDO, Greifensee, Switzerland). Co_2_SiO_4_@DE (−3, −6, and −12) was uniformly mixed with paraffin wax and pressed into a concentric ring with an outer diameter of 7.0 mm, an inner diameter of 3.04 mm, and a thickness of 3.04 mm. Thus, the electromagnetism parameters of Co_2_SiO_4_@DE (−3, −6, and −12), in the frequency range of 2–18 GHz, were obtained by a vector network analyzer (Agilent N5234A, Santa Clara, CA, USA) using the coaxial method.

## 3. Results and Discussion

The X-ray diffraction pattern of Co-precursor@DE-6 is presented in Figure S1. The XRD pattern indicates that the diffraction peaks of Co-precursor@DE-6 are consistent with the standard diffraction pattern of cobalt carbonate hydroxide hydrate (JCPDS card no. 48-0083). Thus, Co_2_SiO_4_@DE-6 was obtained by subsequent calcine. The amount of absorbent is important for the wave-absorbing performance of the sample. Therefore, different loads of Co_2_SiO_4_@DE (corresponding to Co_2_SiO_4_@DE-3, Co_2_SiO_4_@DE-6, and Co_2_SiO_4_@DE-12) were obtained by controlling the hydrothermal time to control the load of Co-precursor.

**Figure 2:**
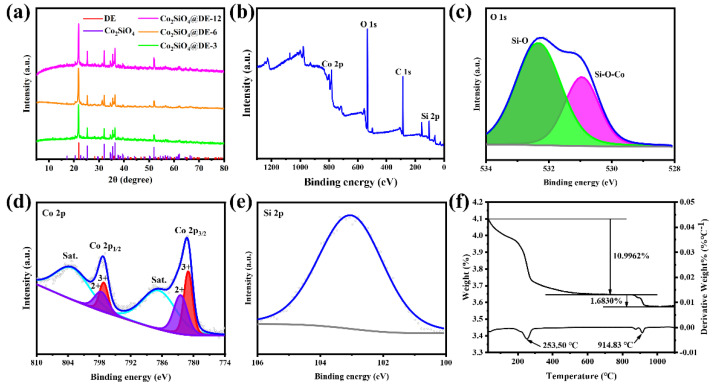
The XRD patterns (**a**) of Co_2_SiO_4_@DE-3, Co_2_SiO_4_@DE-6, and Co_2_SiO_4_@DE-12. The XPS (**b**–**e**) of Co_2_SiO_4_@DE-6: (**b**) full spectrum, (**c**) O 1s, (**d**) Co 2p, and (**e**) Si 2p. The TGA (**f**) of Co-precursor@DE-6.

The X-ray diffraction patterns of Co_2_SiO_4_@DE-3, Co_2_SiO_4_@DE-6, and Co_2_SiO_4_@DE-12 are shown in Figure 2a. The diffraction peaks located at 25.310°, 32.053°, 34.549°, 35.451°, 36.357°, and 52.004° may be assigned to the (111), (031), (220), (131), (211), and (222) crystal planes of Co_2_SiO_4_ (JCPDS card no. 15-0865). Other diffraction peaks located at 21.891°, 31.265°, and 35.995° may be assigned to the (101), (102), and (200) of diatomite (JCPDS card no. 76-0937). Apart from the diffraction peaks of Co_2_SiO_4_ and diatomite, no other impurity diffraction peaks were found in the XRD pattern of Co_2_SiO_4_@DE-3, Co_2_SiO_4_@DE-6, and Co_2_SiO_4_@DE-12. This indicates that the way of the hydrothermal method combined with calcination can obtain purer Co_2_SiO_4_@DE.

Furthermore, the species and valence distribution of the elements of Co_2_SiO_4_@DE-6 were verified by the XPS spectrum. Figure 2b shows the full spectrum of the sample. The sample only contains Co, Si, and O elements. The carbon peak located at 284.6 eV usually is derived from the surroundings [30]. Meanwhile, it proves no other impurity in the sample. The O atom has two states that are only connected with a Si atom and connected together with both a Si atom and Co atom. Therefore, the O element peaks mainly come from the Si–O bond (532.34 eV) in diatomite and Si–O–Co bond (530.95 eV) in Co_2_SiO_4_ (Figure 2c). The transition metal element Co is of more complex XPS peaks due to its more complicated outer electron configuration. The Co element only exists in Co_2_SiO_4_, which has two valences of divalent and trivalent [30]. The Co 2p_1/2_ and Co 2p_2/3_ peaks are composed of Co^2+^–O bonds (797.6 eV and 782.4 eV) and Co^3+^–O bonds (797.1 eV and 781.0 eV) (Figure 2d). The Si atom is only connected with the O atom and therefore the Si element peak is from the Si–O bond (103.0 eV; Figure 2e).

Figure 2f presents the mass loss steps of the Co-precursor@DE-6 in the process of increasing the temperature from 30 °C to 1100 °C. Two mass loss steps are observed at 253.50 °C and 914.83 °C with mass losses of 10.9962% and 1.6830%. The phenomena may indicate that the formation of Co_2_SiO_4_@DE includes three processes of dehydroxylation, decarbonation, and reaction. Therefore, the chemical reactions inferred are presented in Equations (1) and (2):

(1) $${3\mathrm{Co}}_{2}{\mathrm{CO}}_{3}{\left(\mathrm{OH}\right)}_{2}+O_{2}\overset{\Delta }{\to }{2\mathrm{Co}}_{3}O_{4}+{3\mathrm{CO}}_{2}↑+{3H}_{2}O↑$$

(2) $${2\mathrm{Co}}_{3}O_{4}+{3\mathrm{SiO}}_{2}\overset{\Delta }{\to }{3\mathrm{Co}}_{2}{\mathrm{SiO}}_{4}+O_{2}↑$$

**Figure 3:**
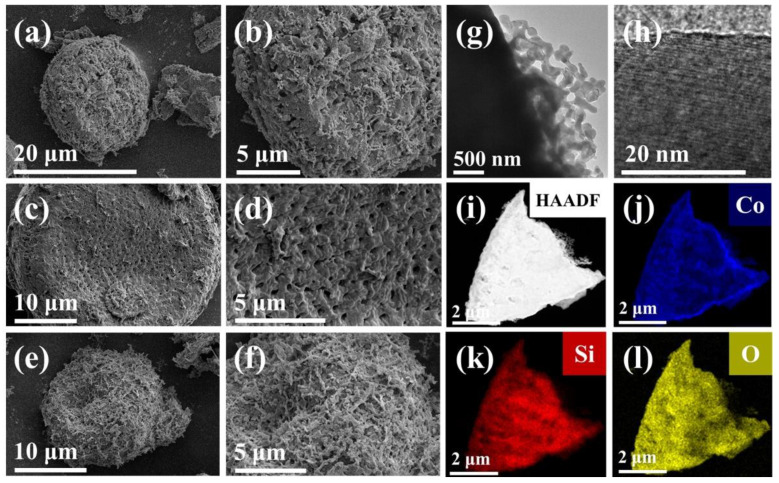
The SEM images of (**a**,**b**) Co_2_SiO_4_@DE-3, (**c**,**d**) Co_2_SiO_4_@DE-6, and (**e**,**f**) Co_2_SiO_4_@DE-12. The TEM images (**g**–**l**) of Co_2_SiO_4_@DE-6: (**g**,**h**) TEM mode, (**i**) HADDF mode, and (**j**–**l**) EDS mapping mode.

The SEM images of the micro-morphology of Co_2_SiO_4_@DE-3, Co_2_SiO_4_@DE-6, and Co_2_SiO_4_@DE-12 are presented Figure 3a–f. Diatomite is of a porous structure, which can increase the multiple reflection and absorption of incident electromagnetic waves to improve the wave-absorbing performance. As shown in Figure 3a–f, Co_2_SiO_4_ loaded on diatomite significantly increases the surface roughness of diatomite, which is beneficial to diffuse the reflection of the incident electromagnetic wave. Furthermore, the load of Co_2_SiO_4_ increasing with the hydrothermal time increasing from 3 h to 12 h was observed. The optimum hydrothermal time was determined by the subsequent measure of the wave-absorbing performance. The TEM image (Figure 3g) indicates that the morphology of calcined Co_2_SiO_4_ may be strip-shaped The high-resolution TEM image (Figure 3h) shows the lattice stripes of the material, which indicates that the material is crystal. The result is consistent with the XRD image. Furthermore, the high-resolution TEM image and EDS mapping (Figure 3i–l) further prove that the sample preparation is successful and Co_2_SiO_4_ is uniformly as well as completely coated with diatomite.

**Figure 4:**
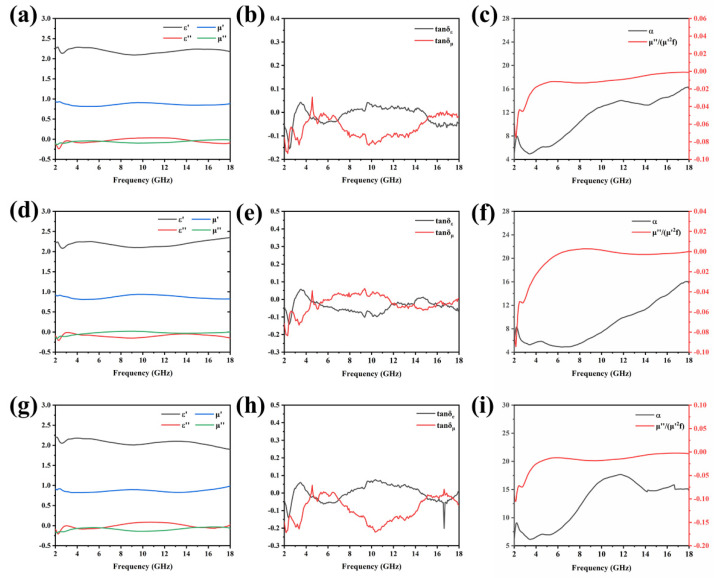
Relevant EM parameters of Co_2_SiO_4_@DE-3 (**a**–**c**), Co_2_SiO_4_@DE-6 (**d**–**f**), and Co_2_SiO_4_@DE-12 (**g**–**i**). Frequency dependence of $\varepsilon^{\prime },\varepsilon^{\prime \prime },\mu^{\prime }$, and $\mu^{\prime \prime }$ (**a**,**d**,**g**); dielectric and loss tangent (**b**,**e**,**h**); and attenuation constant α and $\mu^{\prime \prime }{\left(\mu^{\prime }\right)}^{2}f^{-1}$ values (**c**,**f**,**i**).

In this experiment, a vector network analyzer was used to obtain the electromagnetic parameters of the material, including the real and imaginary parts of the complex permittivity and permeability. These data can directly indicate whether the material is suitable as EMWAM. The results of the characteristics are shown in Figure 4. Due to the unique hollow and porous structure of pure diatomite, it exhibits a good dielectric property. As shown in Figure 4, whether the material is calcined or not, the real part of its complex permittivity is around 2.5 and the imaginary part is around 1. This shows that the loading of Co_2_SiO_4_ will not affect the dielectric properties of diatomite. On the other hand, the imaginary part of the magnetic permeability (μ″) represents the loss modulus of magnetic energy. The μ″ value of the diatomite loaded with Co_2_SiO_4_ is higher than the material without calcination and this shows that the EMW absorption ability of calcined diatomite is enhanced. This change may be due to the increase in the specific surface area of the material, resulting in more interface polarization.

On the basis of electromagnetic parameters, the dielectric loss tangent (ε″/ε′) value and the magnetic loss tangent (μ″/μ′) value (Figure 4) were calculated further. These two values can indicate the absorption performance of the material from the other point of view. In addition to the loss tangent, the attention constant (α) is also an important parameter. α indicates the material’s ability to attenuate EMW. The larger the value, the stronger the EMW dissipate ability of the material. The specific calculation formula is as follows [31]:

(3) $$\alpha =\frac{\sqrt{2}\pi f}{c}\sqrt{\left(\mu^{\prime \prime }\varepsilon^{\prime \prime }-\mu^{\prime }\varepsilon^{\prime }\right)+\sqrt{\left(\mu^{\prime \prime }\varepsilon^{\prime \prime }-\mu^{\prime }\varepsilon^{\prime }\right){}^{2}+\left(\mu^{\prime }\varepsilon^{\prime \prime }+\mu^{\prime \prime }\varepsilon^{\prime }\right){}^{2}}}$$

where f is the frequency of the incident electromagnetic wave and c is the speed of the EMW in vacuum (considered as 3 × 10^8^). It can be seen from the gray data line in Figure 4c–i that as the frequency increases from 2 GHz to 18 GHz, the α values of the three samples show the same trend. The three α reach around 16 at 18 GHz. Therefore, it can be inferred that the three samples obtain a better EMW absorption effect at the maximum frequency of 18 GHz.

On the basis of analyzing the changes of the electromagnetic parameters with frequency, the actual influence of materials on EMW absorption was mainly studied. In order to show the absorption effect more intuitively, the concept of the reflection loss (RL) value was introduced. The RL value is usually a negative value and the lower the RL value, the better the EMW absorption property of the material. Generally speaking, when RL is lower than −10 dB, it means that 90% of incident EMW will be converted into heat or other forms of energy. The frequency range that meets the above conditions is usually defined as the effective absorption bandwidth (EAB). The RL calculation formulas are as follows [32,33]:

(4) $$\mathrm{RL}\;\left(\mathrm{dB}\right)=20\log\left|\left(Z_{\mathrm{in}}-Z_{0}\right)/\left(Z_{\mathrm{in}}{+Z}_{0}\right)\right|$$

(5) $$Z_{\mathrm{in}}=Z_{0}\sqrt{\mu_{r}/\varepsilon_{r}}\tan h\left[j\left(2\pi \mathrm{fd}/c\right)\sqrt{\mu_{r}\varepsilon_{r}}\right]$$

where $Z_{0}=\sqrt{\mu_{0}/\varepsilon_{0}}$ is the characteristic impedance of free space; $Z_{\mathrm{in}}$ is the input impedance at the free space and material interface; and f, d, and c are the frequency of microwaves in free space, the thickness of the absorber, and the velocity of light, respectively.

**Figure 5:**
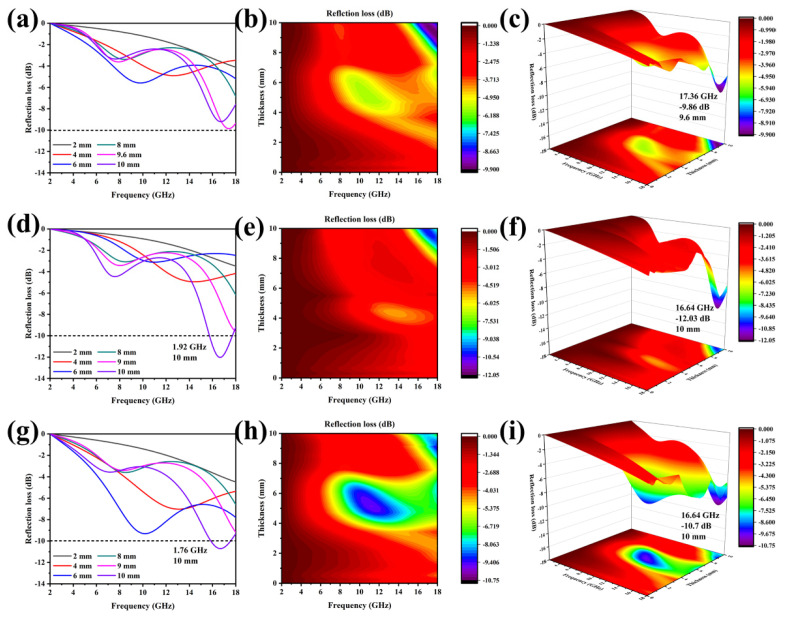
One-dimensional, 2D, and 3D diagram of the RL value changing with frequency and thickness: (**a**–**c**) Co_2_SiO_4_@DE-3, (**d**–**f**) Co_2_SiO_4_@DE-6, and (**g**–**i**) Co_2_SiO_4_@DE-12.

In Figure 5, the first-to-third rows’ figures represent the RL values of Co_2_SiO_4_@DE (−3, 6, and 12) and the three columns from left to right are the 1D, 2D, and 3D graphs of the RL value of the three samples as a function of frequency and thickness. By the hydrothermal and calcination process, diatomite and Co_2_SiO_4_ combine to form a double-shell structure, which is conducive to the EMW absorption. For the Co_2_SiO_4_@DE-3, in the range of 2–18 GHz, even if the thickness is adjusted, the RL value cannot reach −10 dB (Figure 5a–c), which indicates that Co_2_SiO_4_@DE-3 has no EMW absorption ability and can only be used to adjust the impedance matching of the composite. Considering Co_2_SiO_4_ has the potential to become an EMWAM, when the load is increased, it shows a certain EMW absorption performance. When the thickness of Co_2_SiO_4_@DE-6 is 10 mm, its total effective absorption bandwidth in the high frequency range (15.3–17.22 GHz) is 1.92 GHz and the minimum reflection loss is −12.03 dB. When the thickness of Co_2_SiO_4_@DE-12 is 10mm, the effective absorption bandwidth reaches 1.76 GHz and the minimum reflection loss can reach −10.7 dB. It can be seen that when the hydrothermal time is 6 h, the EMW absorption performance is the best. As the hydrothermal time increases, the EMW absorption performance tends to deteriorate. This may be the reason why when the loading of Co_2_SiO_4_ increases to a certain extent, the Co_2_SiO_4_ fully covers the porous structure of diatomite. This leads to an obstruction of the transmission of dielectric signals and to a certain negative influence on the reflection and refraction of EMW.

The Eddy current loss is an important source of magnetic loss and in this experiment, the magnetic Co_2_SiO_4_ played the role of the magnetic loss component. According to Figure 4, the influence of the Eddy current loss on the EMW absorption property was analyzed through calculation preliminarily. Equation (6) was used to judge the effect [34]:

(6) $$\mu^{\prime \prime }{\left(\mu^{\prime }\right)}^{2}f^{-1}={2\pi \mu }_{0}d^{2}\sigma$$

The right side of the equation is a constant. If the magnetic loss is only the Eddy current loss, the magnetic permeability parameter is within the frequency range and the material will satisfy the formula. It can be seen that in the range of 2–18 GHz, the value of $\mu^{\prime \prime }{\left(\mu^{\prime }\right)}^{2}f^{-1}$ fluctuates around 0 rather than being a horizontal line. Therefore, it can be inferred that the Eddy current loss is not the only cause of magnetic loss. Other important reasons such as hysteresis loss, natural resonance, and exchange resonance can also cause the magnetic loss of materials.

**Figure 6:**
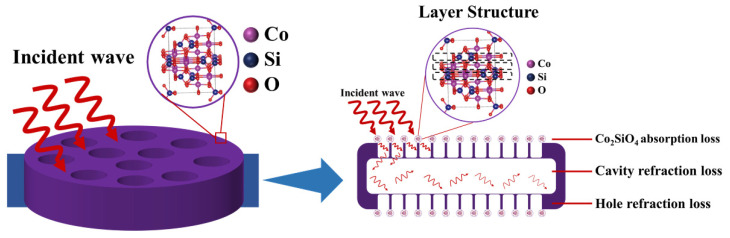
Wave absorption mechanism of Co_2_SiO_4_@DE composite material.

In addition to magnetic loss, dielectric loss is also an important reason for good EMW absorption property. When EMW is an incident on a dielectric material, the first microscopic mechanism encountered is the polarization of the medium, which is very different from the polarization of a conductor with free electrons. For diatomite, the electrons are in a bound state under the action of an alternating electric field. The silicate molecules produce dielectric polarization and the central position of the positive and negative charges changes from superposition to separation. This phenomenon will generate a rotational torque when an electric dipole and a weak electric field is formed. Therefore, the dielectric loss of the material mainly comes from the rotation and orientation of the dipole during the polarization process. Secondly, it comes from the resonance when the frequency of the external electric field is consistent with the molecular thermal vibration frequency. Consequently, the absorbing principles of Co_2_SiO_4_@DE were analyzed in detail in Figure 6. First of all, when EMW is an incident on the EMWAM, the Co_2_SiO_4_@DE makes most of the electromagnetic energy smoothly enter the absorbing material by a porous and layered absorber with impedance matching adjustment instead of reflecting on the surface. Then, after loading Co_2_SiO_4_, the surface roughness of diatomite increases, which will increase the scattering and refraction effects of EMW. Most importantly, the large cavity and porous structure of diatomite can make the incident wave produce multiple reflections and refractions, prolong the path, and increase the loss of incident waves to absorb most of the incident wave. The structure is not available in many materials, especially in 2D materials. Meanwhile, the interface of the composite also promotes the attenuation of the incident wave. Finally, the dense nano-sheet structure on the inner and outer surfaces of diatomite earth can effectively scatter electromagnetic waves. Therefore, it will cause an EMW incident in the same direction to change the EMW original directions to result in superimposition and cancellation. Meanwhile, the EMW that do not enter the absorbent or escape from the absorbent will be consumed by multiple reflections between the absorbent particles.

Combining the above characterization results and analysis conclusions, it is not difficult to conclude that Co_2_SiO_4_@DE has a good application prospect for EMW absorption.

## 4. Conclusions

In summary, the Co_2_SiO_4_@DE composite material has been successfully synthesized through the one-step hydrothermal method and calcination. Through XRD, SEM, TEM, and other characterization results combined with theoretical analysis, the EMW-absorbing properties of Co_2_SiO_4_@DE obtained by calcination of a precursor with the hydrothermal time of 6 h are best. The minimum reflection loss can reach −12.03 dB at 16.64 GHz and its effective bandwidth is 1.92 GHz. The magnetic hysteresis loss of Co_2_SiO_4_ can make up for the lack of magnetic diatomite. At the same time, the cavity and porous structure of diatomite also promote the EMW absorption performance of the diatomite composite. Furthermore, we will conduct more fine structural regulations on Co_2_SiO_4_@DE to improve the EMW-absorbing performance and make it a diatomite composite material with good application prospects as a novel and lightweight EMWAM.

## Data Availability

Data of the compounds are available from the authors.

## Supplementary Materials

The following supporting information are available online, Figure S1:The XRD patterns of Co-precursor@DE-6 and Co_2_SiO_4_@DE-6.

## Sample Availability

Samples of the compounds are available from the authors.
