# Supplementary material for: Lightweight, Low-Cost Co2SiO4@diatomite Core-Shell Composite Material for High-Efficiency Microwave Absorption
Source: Molecules. 2022 Feb 5;27(3):1055. doi: 10.3390/molecules27031055 (PMC8838198; doi:10.3390/molecules27031055)
Supplement: Supplementary file 1 [file molecules-27-01055-s001.zip › molecules-1555859-supplementary.pdf]

Supporting Information

Lightweight, Low-Cost Co_2SiO_4@diatomite Core-shell Composite Material for High-Efficiency Microwave Absorption

Yifan Zhang[1, †], Rui Cai[2, †], Dashuang Wang[1], Kailin Li[1], Qing Sun[3], Yuntao Xiao[1], Hao Teng[1], Xiaohan Huang[4], Tao Sun[4], Zhaohui Liu[5], Kexin Yao[5], Yuxin Zhang[1, *], Pingan Yang[2, *]

[1] College of Material Science and Engineering, Chongqing University, Chongqing 400044, China; zhangyifan@cqu.edu.cn (Y. Z.); Waloneds@sina.com (D. S.); likailin920809@163.com (K. L.); 20202732@cqu.edu.cn (Y. X.); 20192966@cqu.edu.cn (H. T.)

[2] School of Automation, Chongqing University of Posts and Telecommunications, Chongqing 400065, China;

crrrui@163.com (R. C.)

[3] College of Chemistry and Chemical Engineering, Chongqing University, Chongqing 400045, China;

20160902049@cqu.edu.cn (Q. S.)

[4] Department of Military Facilities, Army Logistics University of PLA, Chongqing 401311, China; hxhshr@sina.com (X. H.); suntao_tju@126.com (T. S.)

[5] Multi-scale Porous Materials Center, Institute of Advanced Interdisciplinary Studies & School of Chemistry and Chemical Engineering, Chongqing University, Chongqing 400045, China; zhaohui.liu@cqu.edu.cn (Z. L.); kexinyao@cqu.edu.cn (K. Y.)

[*]Author to whom correspondence should be addressed.

[*] Correspondence: zhangyuxin@cqu.edu.cn (Y. Z.); yangpa@cqupt.edu.cn (P. Y.)

[†] These authors contributed equally to this work.

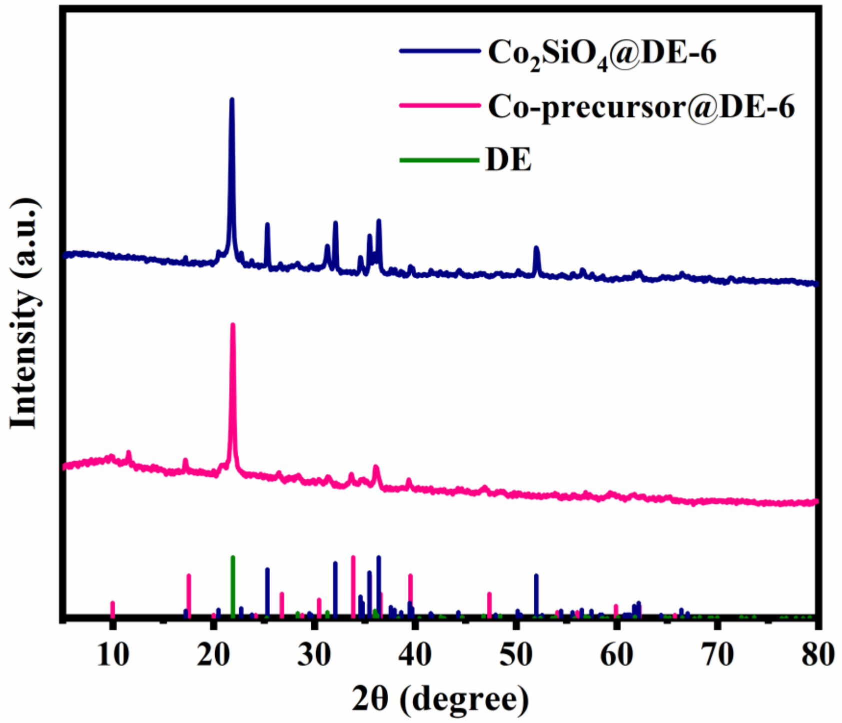

Figure S1. The XRD patterns of Co-precursor@DE-6 and Co_2SiO_4@DE-6.
